# Association Between TNF-α-308, +489, −238 Polymorphism, and COPD Susceptibility: An Updated Meta-Analysis and Trial Sequential Analysis

**DOI:** 10.3389/fgene.2021.772032

**Published:** 2022-01-21

**Authors:** Zhiyu Xia, Yufei Wang, Fu Liu, Hongxin Shu, Peng Huang

**Affiliations:** ^1^ The Second Clinical Medical School, Nanchang University, Nanchang, China; ^2^ Center for Evidence-Based Medicine, School of Public Health, Nanchang University, Nanchang, China; ^3^ Jiangxi Province Key Laboratory of Preventive Medicine, School of Public Health, Nanchang University, Nanchang, China

**Keywords:** COPD, single nucleotide polymorphism, meta-analysis, trial sequential analysis, TNF-α

## Abstract

The tumor necrosis factor alpha (TNF-α) polymorphism may play an important role in chronic obstructive pulmonary disease (COPD) susceptibility. However, the results are still inconclusive. Eligible studies were searched in Cochrane Library database, EMBASE, Pudmed, Web of science, China National Knowledge Infrastructure, and Wanfang database. Finally, a total of 27 case-control studies with 3473 COPD cases and 4935 controls were included in the present analysis. We also performed trial sequential analysis (TSA) to confirm our results. Overall, association between TNF-α-308G/A polymorphism and COPD susceptibility was identified in allelic model (A vs. G, OR = 1.21, 95%CI: 1.01–1.45, *p* = 0.04) when smoking status was not adjusted. In ethnicity subgroup analysis, we found that the TNF-α -308G/A polymorphism was associated to COPD among Asians (GA vs. GG, OR = 1.35, 95%CI: 1.04–1.77, *p* = 0.02) when smoking status was not adjusted. However, no significant association was found in Asian smokers or Caucasian smokers. In conclusion, our study suggest that TNF-α-308 GA genotype is related to COPD in the Asian population. In addition, the TNF-α+489G/A, - 238G/A variants do not increase the risk of COPD.

**Systematic Review Registration**: https://www.crd.york.ac.uk/PROSPERO/, identifier CRD42021273980.

## Introduction

Chronic obstructive pulmonary disease (COPD) is characterized by progressive airflow obstruction and bronchial hyperresponsiveness, with increasing morbidity, mortality, and resource utilization worldwide (Celli et al., 2015). According to the latest figures, COPD has been estimated to become the fourth leading cause of death in the world by 2040 (Foreman et al., 2018).

Tumor necrosis factor alpha (TNF-α) gene is found on chromosome 6p21.33 (Nedwin et al., 1985). The TNF-α-308 G/A polymorphism appears to affect the multifunctional proinflammatory cytokine (Zhou et al., 2016). The signals are mediated through two transmembrane receptors, TNFR1 and TNFR2, to regulate the inflammatory cell functions such as cell proliferation, survival, differentiation, and apoptosis (Parameswaran and Patial, 2010). Some epidemiologic research on the association between TNF-α+489 G/A and -308 G/A polymorphism and COPD susceptibility have been performed (Cui et al., 2015; Zhang et al., 2016; Reséndiz-Hernández et al., 2018; Yu et al., 2021), but the results are still inconsistent. Additionally, the scarcity of meta-analysis about the relationship between TNF-α-238G/A and COPD risk impels us to study the issue in this article. In order to do this, we pooled 27 original studies and followed a stricter criterion to obtain an explicit understanding. Subgroup analysis based on ethnicity was also performed to gain a comprehensive view.

## Materials and Methods

We followed the PRISMA guidelines and registered the review protocol on PROSPERO (CRD42021273980, September 21, 2021).

### Literature Retrieval

A comprehensive search of PubMed, EMBASE, Web of science, Cochrane Library database, Wanfang databases, and China National Knowledge Infrastructure were performed to find eligible studies. We retrieved articles using search strategies: “TNF-α or Tumor Necrosis Factor-α” and “COPD or COAD or chronic obstructive lung disease or chronic obstructive pulmonary disease or chronic obstructive airway disease” and “polymorphism or SNP or genotype or variant” published before July 25, 2021. In addition, we reviewed all references cited in the identified articles.

### Inclusion and Exclusion Criteria

The included studies had to meet all four criteria: (Celli et al., 2015): Case-control or cohort studies, (Foreman et al., 2018), Availability of sufficient information to the odds rations (ORs) with 95% confidence intervals (CIs), (Nedwin et al., 1985), Alleles or genotypes frequencies in control groups and case groups could be received from the studies, and (Zhou et al., 2016) Literature in English. Accordingly, the exclusion criteria were as follows: (Celli et al., 2015): Results not focused on TNF-α-308, +489, or −238; (Foreman et al., 2018); Meta-analyses, letters, reviews, editorial articles, and studies that duplicated previous publications; (Nedwin et al., 1985); Detailed genotype data were not provided; or (Zhou et al., 2016) the genotype distribution of control was not in accordance with the Hardy-Weinberg equilibrium (HWE).

### Data Extraction and Literature Quality Evaluation

Two investigators retrieved data from the included studies separately, and all discordances were discussed to reach an agreement. The extraction information mainly includes the first author’s name, year of publication, country of origin, ethnicity, source of control, genotype method, the overall number of cases and controls, and genotype distribution of three TNF-α gene variants. The Newcastle-Ottawa Scale (NOS) was applied to estimate the quality of included articles, and the studies were classified as high quality (scores ≥7 stars) or low quality (scores<7 stars).

### Statistical Analyses

The odds ratio (OR) and its 95% confidence interval (95%CI) were used as an effect size to assess the risk of COPD caused by TNF-α polymorphism, as well as the strength of association between them. Z-test was used to estimate the statistical significance of pooled ORs. There were five genetic comparison models: allelic model (A vs. G), heterozygote model (GA vs. GG), homozygote model (AA vs. GG), dominant model (GA + AA vs. GG), and recessive model (AA vs. GG + GA). We checked the heterogeneity assumption by the Q-test. The outcome (*p* > 0.1 and I^2^<50%) indicated no heterogeneity among studies, and fixed-effects model (the Mantel-Haenszel method) was applied. Otherwise, when I^2^ ≥ 50% or *p* ≤ 0.10, we performed random-effects model (the DerSimonian and Laird method). Publication bias of the literature was evaluated by funnel plots and Egger’s test, and one-way sensitivity analyses were performed to assess the stability of the results.

The pooled OR and its 95%CI were calculated by Review Manager 5.4.1 software. All the *p*-values are two-sided.

### Trial Sequential Analysis

We used the TSA v0.9.5.10 Beta software to perform the trial sequential analysis. Our study set the relative risk reduction to 20%, the first type of error *α* = 0.05, and power = 80% to evaluate required information size (RIS) and the trial sequential monitoring boundary (TSMB). The results are considered reliable and stable when the Z-value crosses TSMB. At the same time, the sample size can be deemed adequate. However, if the cumulative Z value does not cross the TSMB or RIS threshold, it means the sample size is not sufficient. And it still needs more studies to confirm the result.

## Results

### Features of Recruited Studies

**FIGURE 1:**
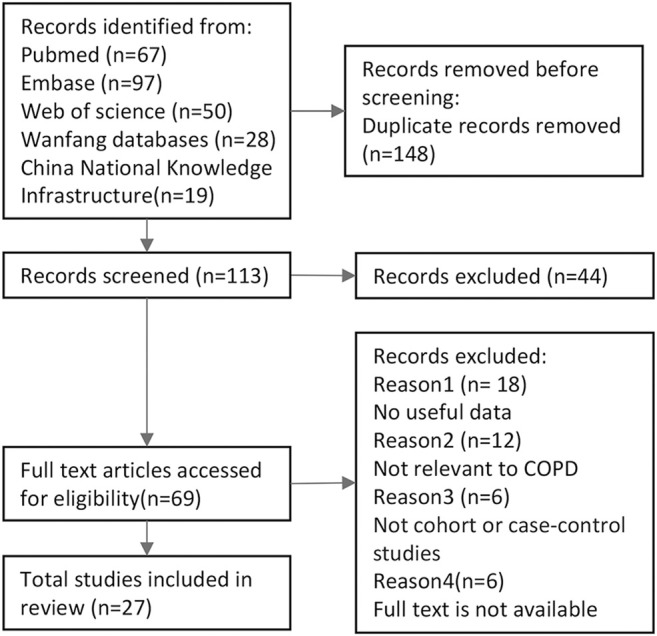
Flowchart illustrating the search strategy for TNF-α polymorphism. and the risk of COPD.

The search strategy yielded 261 potentially relevant articles, and 27 publications with 3473 COPD cases and 4935 controls were ultimately included in the present analysis (Figure 1). All the cases were confirmed by the diagnostic criteria of COPD. There were 25 articles that studied the TNF-α-308 variant, five articles on the TNF-α+489 variant, and four articles on the TNF-α-238 variant. These studies fell into two groups: 16 Asian articles and 20 Caucasian articles. The genotype distributions of TNF-α were found to be in the Hardy-Weinberg equilibrium. Smoking status was adjusted for in 10 studies, and these were included for the second meta-analysis (Table 1).

**TABLE 1:** Main characters of studies included in this meta-analysis.

0.5	Country	Ethnicity	Source of control	Genotype methods	Cases/Controls	Genotype distribution									NOS	HWE
						Case				Control						
						TNF-α 308										
						Smoking status	GG	GA	AA	Smoking status	GG	GA		AA		
(Keatings et al., 2000)	Britain	Caucasian	HB	TaqMan	106/99	Yes	62	38	6	Yes	59	37		3	6	0.871
(Higham et al., 2000)	Britain	Caucasian	HB	RCR-RFLP	86/262	Yes	62	22	2	Mixed	181	74		7	8	0.998
					86/63					Yes	45	17		1	8	<0.05
(Ishii et al., 2000)	Japan	Asian	HB	PCR	53/65	Yes	52	1	0	Yes	64	1		0	7	1.000
(Sakao et al., 2001)	Japan	Asian	HB	PCR- RFLP	106/239	Yes	77	23	6	Mixed	209	22		8	7	0.09
					106/110					Yes	96	10		4	7	<0.05
(Küçükaycan et al., 2002)	Netherlands	Caucasian	PB	PCR	163/335	Mixed	113	49	1	Unknown	237	91		7	6	0.589
(Ferrarotti et al., 2003)	Italy	Caucasian	HB	PCR	63/86	Mixed	54	9	0	Yes	72	14		0	6	0.578
(Chierakul et al., 2005)	Thailand	Asian	HB	PCR	57/183	Yes	48	9	0	Mixed	162	21		0	6	0.585
					57/67					Yes	57	10		0	7	0.598
(Hegab et al., 2005)	Japan	Asian	HB	PCR-RFLP	88/61	Yes	86	2	0	Yes	61	0		0	6	1.000
		Caucasian	HB	PCR-RFLP	106/72	Yes	91	14	1	Yes	57	14		1	6	0.839
(Seifart et al., 2005)	Germany	Caucasian	PB	PCR	113/243	Mixed	73	36	4	Mixed	171	67		4	6	0.857
(Jiang et al., 2005)	China	Asian	PB	PCR-RFLP	101/96	Mixed	90	10	1	Mixed	90	6		0	7	0.597
(Papatheodorou et al., 2007)	Greece	Caucasian	PB	PCR	116/202	Mixed	101	14	1	Yes	169	29		4	6	0.429
(Danilko et al., 2007)	Russia	Asian	HB	PCR	319/381	Unknown	216	101	2	Unknown	273	103		5	7	0.071
(Trajkov et al., 2009)	France	Caucasian	HB	PCR-SSP	60/301	Mixed	45	14	1	Unknown	231	66		4	8	0.952
(Gingo et al., 2008)	America	Caucasian	PB	PCR	298/125	Yes	220	67	11	Yes	105	18		2	7	0.283
(Hsieh et al., 2008)	America	Asian	HB	PCR-RFLP	30/115	Mixed	23	6	1	Mixed	96	18		1	7	0.838
(Stankovic et al., 2009)	Serbia	Caucasian	PB	PCR-RFLP	97/102	Mixed	79	17	1	Mixed	71	28		3	6	0.996
(Chen et al., 2010)	China	Asian	HB	PCR-RFLP	145/139	Yes	117	28	0	Yes	109	27		3	7	0.996
(Shukla et al., 2012)	India	Asian	HB	ARMS-PCR	208/204	Mixed	178	30	0	Mixed	159	41		4	6	1.000
(Tarigan et al., 2015)	Indonesia	Asian	HB	PCR-RFLP	93/93	Yes	75	8	10	Yes	60	25		8	6	<0.05
(Yang et al., 2014)	China	Asian	HB	PCR-RFLP	101/80	Mixed	73	25	3	Mixed	71	9		0	7	0.878
					74/46	Yes	53	19	2	Yes	42	4		0	7	0.991
(Özdoğan et al., 2014)	Turkey	Caucasian	HB	PCR	60/30	Yes	44	16	0	Yes	24	6		0	6	0.543
(Akparova et al., 2017)	India	Asian	PB	PCR	55/52	Mixed	37	13	5	Mixed	36	14		2	7	0.477
(Reséndiz-Hernández et al., 2018)	Mexico	Caucasian	PB	PCR	384/674	Yes	342	42	0	Yes	641	31		2	8	0.844
(Umi Partan and Hidayat, 2019)	Indonesia	Asian	HB	PCR-RFLP	70/35	Unknown	52	17	1	Unknown	27	8		0	6	0.837
(Mir et al., 2020)	India	Asian	HB	PCR	100/163	Mixed	91	9	0	Mixed	150	13		0	7	0.989
(Yu et al., 2021)	China	Asian	HB	PCR	198/195	Mixed	173	21	4	Unknown	186	9		0	7	0.308
—						TNF-α 489									—	—
						Smoking status	GG	GA	AA	Smoking status	GG	GA	AA			
(Küçükaycan et al., 2002)	Netherlands	Caucasian	PB	PCR	157/315	Mixed	118	38	1	Unknown	264	45	6		6	0.805
(Hegab et al., 2005)	Japan	Asian	HB	TaqMan	88/61	Yes	57	26	5	Yes	40	19	2		6	0.939
		Caucasian	HB	TaqMan	106/72	Yes	85	19	2	Yes	58	13	1		7	0.876
(Hsieh et al., 2008)	America	Caucasian	HB	PCR-RFLP	30/115	Mixed	23	7	0	Mixed	79	35	1		7	0.514
(Matokanovic et al., 2012)	Croatia	Caucasian	HB	PCR-RFLP	130/95	Mixed	87	38	5	Mixed	53	39	3		6	0.863
Yu et al., 2021)	China	Asian	HB	PCR	198/195	Mixed	140	46	12	Unknown	148	39	8		7	0.060
—						TNF-α 238									—	—
						Smoking status	GG	GA	AA	Smoking status	GG	GA	AA			
(Küçükaycan et al., 2002)	Netherlands	Caucasian	PB	PCR	164/331	Mixed	151	13	0	Unknown	310	20	1		6	0.993
(Papatheodorou et al., 2007)	Greece	Caucasian	PB	PCR	116/208	Unknown	111	5	0	Yes	197	11	0		6	1.000
(Trajkov et al., 2009)	France	Caucasian	HB	PCR-SSP	60/301	Mixed	53	7	0	Unknown	276	23	2		7	0.819
(Tarigan et al., 2015)	Indonesia	Asian	HB	PCR-RFLP	93/93	Yes	85	4	4	Yes	89	1	3		6	<0.05
(Reséndiz-Hernández et al., 2018)	Mexico	Caucasian	HB	RT-PCR	384/674	Yes	349	31	4	Yes	621	52	1		8	0.472

Abbreviation: HB, hospital-based; PB, population-based.

### Association of COPD Susceptibility With TNF-α-308

The effect of the TNF-α-308G/A polymorphism on COPD was first investigated by pooling 25 studies where smoking status was not adjusted, comprising 3283 COPD cases and 4539 non-COPD controls. It indicated that the A allele was associated with an increased COPD risk in the overall population (A vs. G, OR = 1.21,95% CI: 1.01–1.45, *p* = 0.04), using an allelic model. According to the comparison between heterozygote model (GA vs. GG, OR = 1.22, 95% CI :1.02–1.45, *p* = 0.03) and dominant model (GA + AA vs. GG, OR = 1.22, 95%CI: 1.01–1.48, *p* = 0.04), GA genotype carriers of TNF-α-308 have a higher risk of developing COPD compared to GG carriers.

**FIGURE 2:**
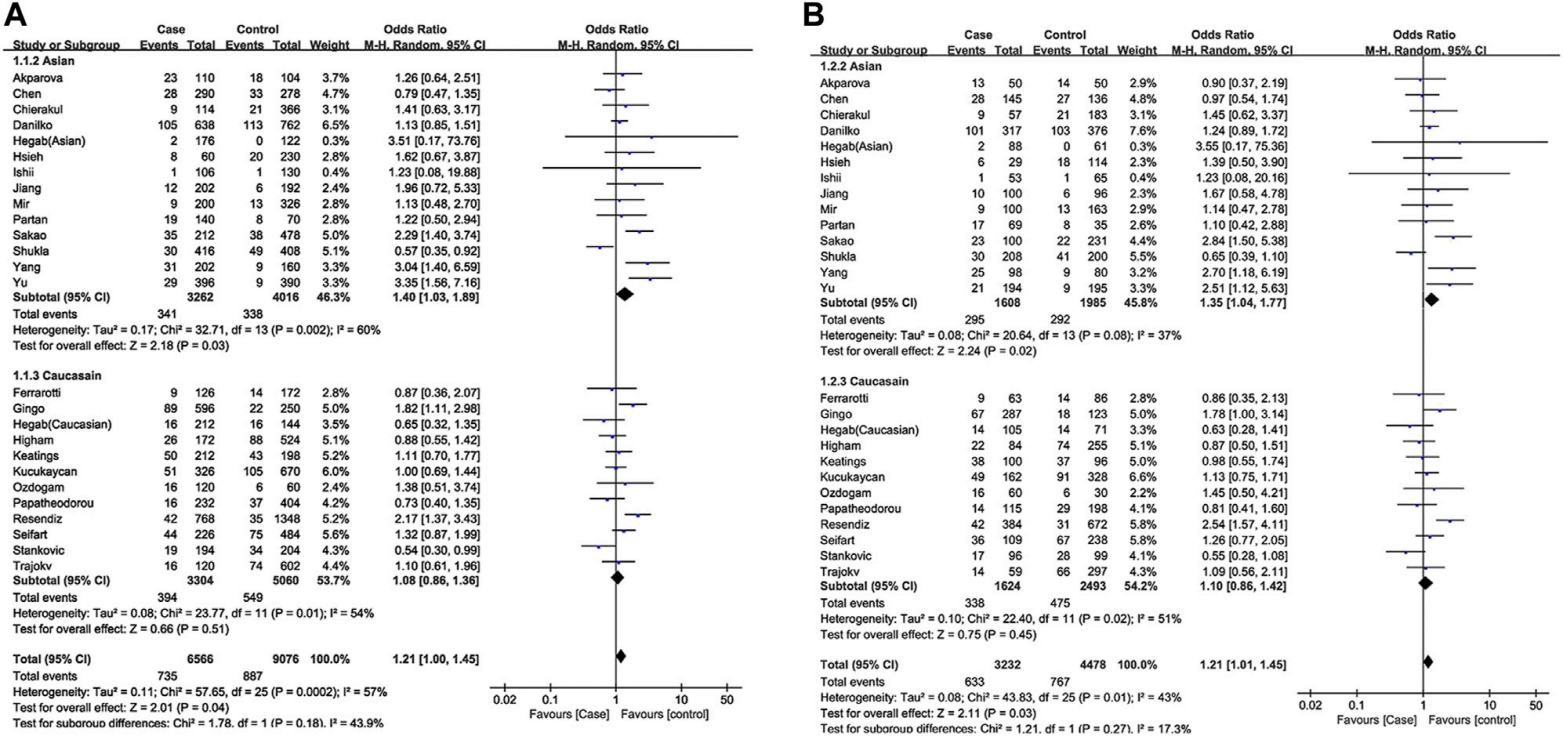
Forest plot of TNF-α-308 polymorphism and COPD risk [**(A)**: overall for A vs. G, **(B)**: overall for GA vs. GG].

In the stratified analysis by ethnicity, we found that the TNF-α-308 G/A polymorphism was associated with COPD risk under the allelic model (A vs. G, OR = 1.40, 95% CI: 1.03–1.89, *p* = 0.03). The pooled OR of heterozygote model was 1.35 (GA vs. GG, OR = 1.35, 95% CI: 1.04–1.77, *p* = 0.02), indicating that the risk of COPD with TNF-α-308 GA genotype was 1.35 times higher than that with the GG genotype in an Asian population. A similar result was also observed in the dominant model (GA + AA vs. GG, OR = 1.40, 95% CI: 1.04–1.88, *p* = 0.03), which further supported our finding. In contrast, no significant association between the TNF-α-308 G/A SNP and COPD was found in Caucasians (Figure 2).

Specific environmental factors, such as smoking, may contribute to the difference in the distribution of genetic polymorphism. Therefore, we conducted a second meta-analysis with 10 studies including 2749 smokers. In this meta-analysis, TNF-α-308 polymorphism was not associated with COPD either in Asian smokers or Caucasian smokers (Table 2).

**TABLE 2:** Meta-analysis results for relationship between the TNF-α-308 polymorphism and COPD risk.; Abbreviation: CI = confidence interval, F = fixed effect model, OR = odds ratio, R = random-effect model.

TNF-α-308		Contrast model	Number of studies	OR (95%CI)	*p*	Test for heterogeneity		Analysis model
						I^2^ (%)	*p*	
Participants included	Overall	A vs. G	25	1.21 (1.01, 1.45)	0.04	57	0.00	R
		GA vs. GG	25	1.22 (1.02, 1.45)	0.03	43	0.01	R
		AA vs. GG	25	1.18 (0.80, 1.73)	0.41	0	0.45	F
		AA vs. GG + GA	25	1.14 (0.77, 1.67)	0.52	0	0.55	F
		GA + AA vs. GG	25	1.22 (1.01, 1.48)	0.04	52	0.00	R
	Asian	A vs. G	14	1.40 (1.03, 1.89)	0.03	60	0.00	R
		GA vs. GG	14	1.35 (1.04, 1.77)	0.02	37	0.08	R
		AA vs. GG	14	1.33 (0.76, 2.33)	0.32	22	0.24	F
		AA vs. GG + GA	14	1.26 (0.72, 2.21)	0.42	17	0.29	F
		GA + AA vs. GG	14	1.40 (1.04, 1.88)	0.03	51	0.01	R
	Caucasian	A vs. G	12	1.08 (0.86, 1.36)	0.51	54	0.01	R
		GA vs. GG	12	1.10 (0.86, 1.42)	0.45	51	0.02	R
		AA vs. GG	12	1.05 (0.61, 1.79)	0.87	0	0.62	F
		AA vs. GG + GA	12	1.03 (0.60, 1.77)	0.91	0	0.69	F
		GA + AA vs. GG	12	1.09 (0.85, 1.41)	0.49	53	0.01	R
Smokers	Overall	A vs. G	9	1.35 (0.97, 1.89)	0.08	52	0.03	R
		GA vs. GG	9	1.41 (0.99, 2.00)	0.06	47	0.06	R
		AA vs. GG	9	1.31 (0.62, 2.75)	0.48	0	0.45	F
		AA vs. GG + GA	9	1.24 (0.59, 2.60)	0.57	0	0.49	F
		GA + AA vs. GG	9	1.40 (0.98, 2.00)	0.06	50	0.04	R
	Asian	A vs. G	5	1.19 (0.80, 1.78)	0.39	47	0.11	F
		GA vs. GG	5	1.32 (0.85, 2.05)	0.21	17	0.30	F
		AA vs. GG	5	0.71 (0.03, 19.93)	0.84	59	0.12	R
		AA vs. GG + GA	5	0.64 (0.03, 14.48)	0.78	53	0.14	R
		GA + AA vs. GG	5	1.27 (0.83, 1.95)	0.27	37	0.17	F
	Caucasian	A vs. G	5	1.38 (0.92, 2.07)	0.12	59	0.05	R
		GA vs. GG	5	1.38 (0.84, 2.27)	0.21	65	0.02	R
		AA vs. GG	5	1.63 (0.68, 3.88)	0.27	0	0.62	F
		AA vs. GG + GA	5	1.56 (0.65, 3.71)	0.32	0	0.65	F
		GA + AA vs. GG	5	1.40 (0.88, 2.23)	0.16	62	0.03	R

### Association of COPD Susceptibility With TNF-α+489

For TNF-α+489G/A variant, five research papers, including 709 cases and 853 normal controls, were selected. The pooled results suggested that TNF-α+489 polymorphism was not associated with COPD risk (*p* > 0.05). Subgroups analysis by ethnicity also showed no association between TNF-α+489 and COPD among Asians (*p* > 0.05) and Caucasians (*p* > 0.05) (Table 3).

**TABLE 3:** Meta-analysis results for relationship between the TNF-α+489, -238 polymorphism and COPD risk.

SNPs	Contrast model	Subgroup	Number of studies	OR (95% CI)	*p*	Test for heterogeneity		Analysis model
						I^2^ (%)	*p*	
TNF-α+489	A vs. G	Overall	5	1.10 (0.89, 1.35)	0.38	41	0.15	F
	GA vs. GG	Overall	5	1.04 (0.68, 1.57)	0.87	63	0.03	R
	AA vs. GG	Overall	5	1.23 (0.66, 2.26)	0.51	0	0.79	F
	AA vs. GG + GA	Overall	5	1.22 (0.67, 2.25)	0.51	0	0.77	F
	GA + AA vs. GG	Overall	5	1.05 (0.73, 1.53)	0.79	57	0.05	R
	A vs. G	Asian	2	1.24 (0.90, 1.71)	0.18	0	0.64	F
	GA vs. GG	Asian	2	1.15 (0.77, 1.72)	0.50	0	0.55	F
	AA vs. GG	Asian	2	1.62 (0.72, 3.65)	0.24	0	0.92	F
	AA vs. GG + GA	Asian	2	1.57 (0.70, 3.50)	0.27	0	0.87	F
	GA + AA vs. GG	Asian	2	1.22 (0.84, 1.77)	0.30	0	0.58	F
	A vs. G	Caucasian	4	1.01 (0.77, 1.32)	0.97	47	0.13	F
	GA vs. GG	Caucasian	4	0.97 (0.52, 1.80)	0.92	71	0.02	R
	AA vs. GG	Caucasian	4	0.81 (0.30, 2.15)	0.67	0	0.84	F
	AA vs. GG + GA	Caucasian	4	0.86 (0.33, 2.23)	0.75	0	0.75	F
	GA + AA vs. GG	Caucasian	4	0.96 (0.56, 1.66)	0.89	65	0.04	R
TNF-α-238	A vs. G	Caucasian	4	1.22 (0.89, 1.67)	0.23	0	0.89	F
	GA vs. GG	Caucasian	4	1.14 (0.81, 1.61)	0.44	0	0.76	F
	AA vs. GG	Caucasian	4	2.64 (0.70, 9.93)	0.15	0	0.40	F
	AA vs. GG + GA	Caucasian	4	2.59 (0.69, 9.69)	0.16	0	0.39	F
	GA + AA vs. GG	Caucasian	4	1.18 (0.85, 1.65)	0.32	0	0.87	F

Abbreviation: CI, confidence interval, F = fixed effect model, OR, odds ratio, R = random-effect model.

### Association of COPD Susceptibility With TNF-α-238

624 cases and 1514 controls originated from four studies were included to probe the relevance between susceptibility to COPD and TNF-α-238G/A variant. We found that TNF-α-238 polymorphism was not associated with COPD risk in the overall population (*p* > 0.05). (Table 3).

### Sensitivity Analyses

**FIGURE 3:**
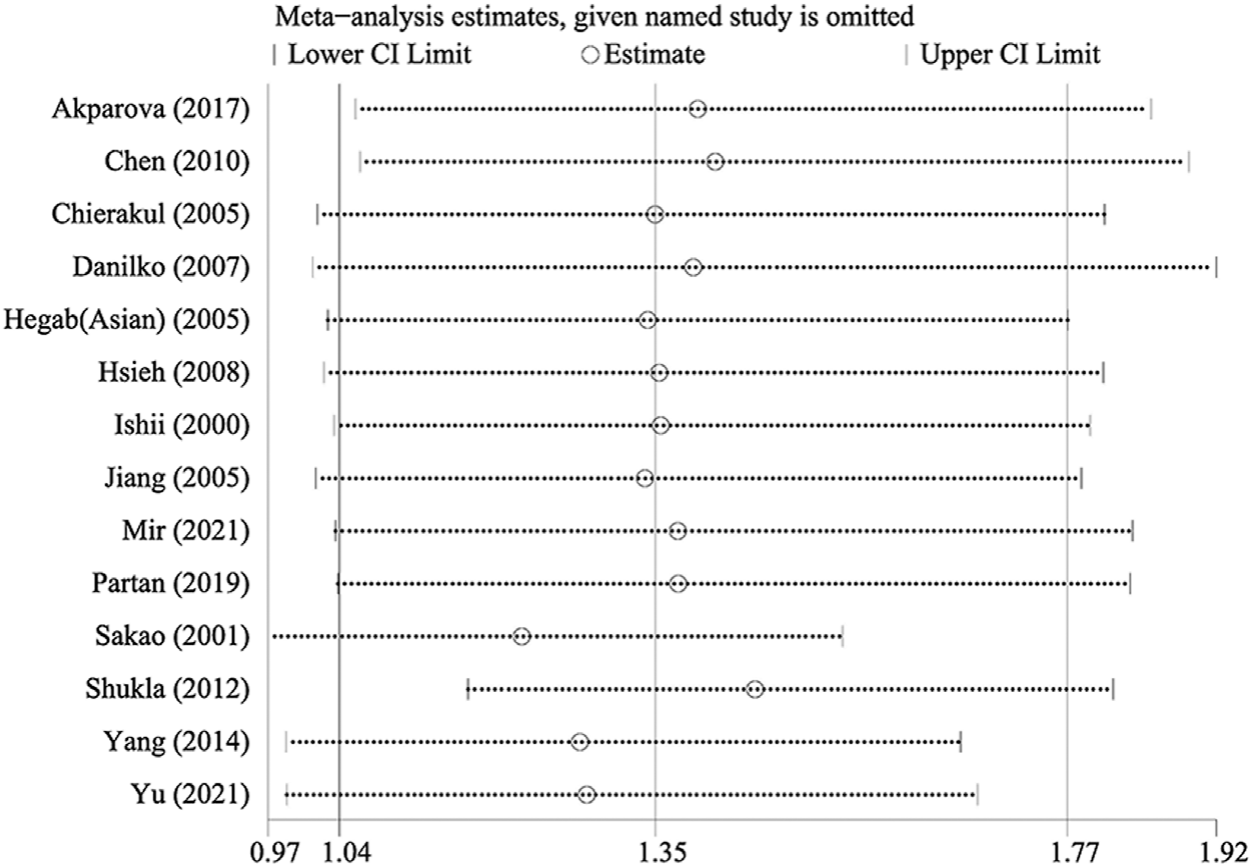
Sensitivity analysis of TNF-α-308 polymorphism and COPD risk (Asian population for GA vs. GG).

Sensitivity analysis was performed by sequentially excluding each study to assess the stability of the results in this meta-analysis. As shown in Table 4, the results based on the overall meta-analysis were not stable (Table 4). However, in the Asian subgroup, the pooled OR did not vary significantly, implying that the findings were reliable (Figure 3). We speculated that the overall stability was affected by those studies restricted to Caucasians.

**TABLE 4:** Sensitivity analysis of the association between TNF-α-308 and COPD risk, under the allelic model in overall population.; Abbreviation: CI = confidence interval, OR = odds ratio.

Author	OR (95%CI)	Z value	*p*	I^2^ (%)
Akparova	1.21 (1.00, 1.46)	1.94	0.05	59
Chen	1.23 (1.02, 1.49)	2.19	0.03	57
Chierakul	1.20 (0.99, 1.45)	1.92	0.06	59
Danilko	1.22 (1.00, 1.48)	1.92	0.05	59
Ferrarotti	1.22 (1.01, 1.47)	2.08	0.04	59
Gingo	1.18 (0.98, 1.43)	1.75	0.08	57
Hegab	1.23 (1.02, 1.48)	2.17	0.03	58
Higham	1.23 (1.02, 1.49)	2.12	0.03	58
Hsieh	1.20 (0.99, 1.45)	1.89	0.06	59
Ishii	1.20 (1.00, 1.45)	2.00	0.05	59
Keatings	1.21 (1.00, 1.47)	1.97	0.05	59
Jiang	1.20 (0.99, 1.44)	1.87	0.06	58
Kucukaycan	1.22 (1.00, 1.49)	2.04	0.04	58
Mir	1.21 (1.00, 1.46)	1.99	0.05	59
Partan	1.21 (1.00, 1.46)	1.97	0.05	59
Resendiz	1.17 (0.97, 1.40)	1.67	0.09	53
Sakao	1.16 (0.97, 1.39)	1.67	0.10	53
Seifart	1.20 (0.99, 1.46)	1.87	0.06	59
Shukla	1.25 (1.05, 1.50)	2.53	0.01	51
Stankovic	1.25 (1.04, 1.49)	2.43	0.02	54
Trajokv	1.21 (1.00, 1.47)	1.99	0.05	59
Ozdogan	1.20 (1.00, 1.45)	1.94	0.05	59
Papatheodorou	1.23 (1.02, 1.49)	2.21	0.03	57
Yang	1.17 (0.97, 1.40)	1.72	0.09	54
Yu	1.16 (0.98, 1.39)	1.69	0.09	53

### Publication Bias

**FIGURE 4:**
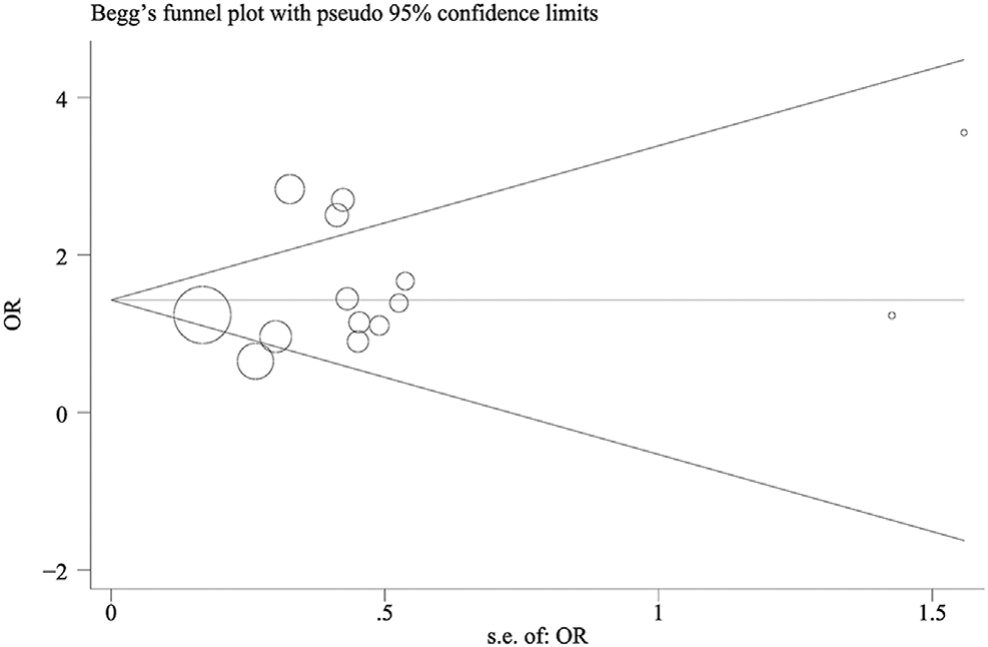
Begg’s funnel plot of TNF-α-308 polymorphism and COPD risk (Asian population for GA vs. GG).

Begg’s funnel plots and Egger’s test were performed to assess the publication bias (all contrast models: *p* > 0.05), and the results suggested that there was no publication bias for the association between TNF-α-308 polymorphism and COPD in included studies (Figure 4).

### Trial Sequential Analysis Results

**FIGURE 5:**
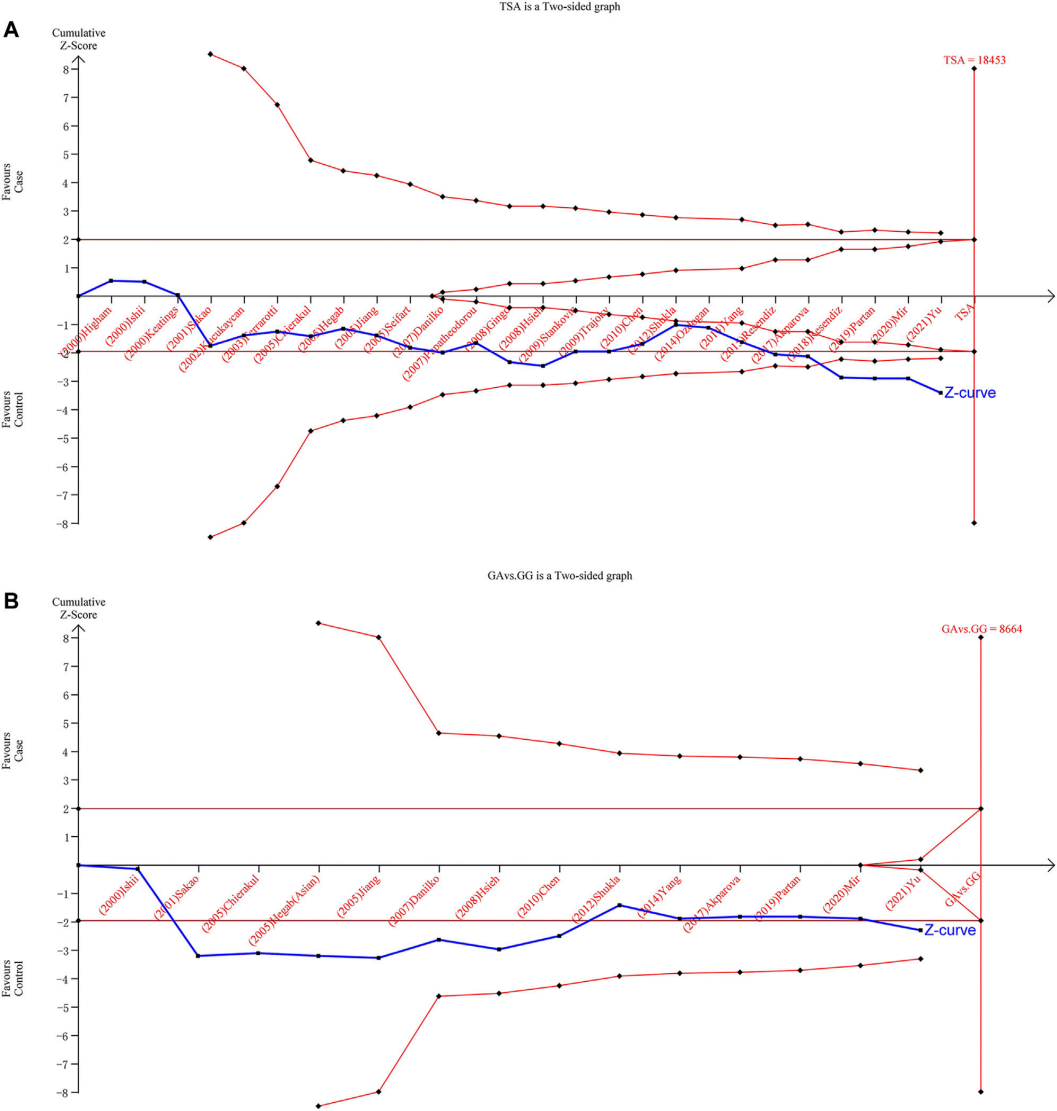
Results of TSA with TNF-α-308 variant. The required information size was calculated based on a two-sided *a* = 5%, *ß* = 15% (power 80%), and a relative risk reduction of 20%. [**(A)**: overall for A vs. G, **(B)**: Asian population for GA vs. GG].

We implemented TSA to reduce the risk of type I error and to evaluate the RIS by keeping the overall 5% risk of the type I error and the relative risk reduction of 20% (power of 80%). The results on TNF-α-308 showed that the Z-curve crossed the TSMB (Figure 5A). Therefore, it can be assumed that mutation from G to A on TNF-α-308 can increase the risk of COPD. The sample size did not reach the RIS among Asians in the allele model, heterozygote model, or dominant model (Figure 5B). Hence, more qualified studies are expected to confirm the relationship between TNF-α-308 variants and COPD susceptibility among Asians.

## Discussion

For the TNF-α-308 G/A, the GA genotype was significantly associated with increased COPD risk, especially among Asians. Presumably, the GA variant was a stronger activator of TNF-α transcription than the GG genotype (Wilson et al., 1997). Some studies had shown that TNF-α-308 GA expression may cause damage to muscle (Baumert et al., 2016) and increase bronchial responsiveness (Moffatt et al., 1999). Moreover, Zhang (Zhang et al., 2016) and other previous studies (Hu et al., 2007; Zhan et al., 2011) found an increased risk of COPD associated with TNF-α-308 AA genotype. Nevertheless, we did not find any significant relationship between COPD susceptibility and TNF-α-308 AA genotype. Our meta-analysis updated five case-control studies (Akparova et al., 2017; Reséndiz-Hernández et al., 2018; Umi Partan and Hidayat, 2019; Mir et al., 2020; Yu et al., 2021) compared with Zhang’s research, and the cumulative Z-value crossed the TSA boundary. The TSA result could be due to the fact that our study had enough COPD cases and controls. This may be the reason for the difference between our analysis and Zhang’s study. Furthermore, we conducted the ethnicity subgroup analysis to gain a more comprehensive view (Salimi Asl et al., 2019; Shi and Zhao, 2019). In our study, a second meta-analysis restricted to smokers was also performed, and it indicated that TNF-α-308G/A polymorphism was not associated with COPD susceptibility among smokers. The result implied that cigarette smoking may conceal the influence of TNF-α polymorphism on COPD. The effect of smoking on the gene factor may be attributed to the following: 1) smoking might exert an interaction with TNF-α-308G/A polymorphism, and have a potential influence on TNF-α gene expression; and 2) smoking is the major risk factor for COPD. We speculated that compared with the effect of TNF-α-308 GA variant on COPD, there was a stronger association between smoking and COPD risk.

As for the TNF-α+489G/A, no significant association was found between TNF-α+489G/A polymorphism and COPD susceptibility. Due to the new case-control study (Yu et al., 2021) included, we proposed a converse conclusion compared with previous meta-analysis (Cui et al., 2015). The result remained controversial because of the limited sample size. Therefore, more case-control studies and cohort studies are in urgent need to determine a stable conclusion.

To the best of our knowledge, this is the first meta-analysis to analyze TNF-α -238G/A variant and its contribution to risk of COPD. Similarly, the pooled results suggested that TNF-α -238G/A polymorphism had no significant association with COPD risk. All the case-control studies about TNF-α-238G/A we retrieved were restricted to Caucasians. Hence, we are looking forward to more original studies on other ethnicities to confirm our conclusions.

Our study features some advantages. Firstly, we performed TSA to reduce the risk of type I error and evaluate required information size. The TSA result showed that the conclusion about TNF-α-308 G/A was based on a sufficient number of cases and controls. Secondly, we carried out a second meta-analysis restricted to smokers. As smoking is the major risk factor for the development of COPD, this sub-group analysis illustrates the additional information genetic polymorphism provides when studying risk factors of COPD. Thirdly, NOS quality test was performed to estimate the quality of the case-control studies included, making the results more reliable. In addition, sensitivity analysis and Egger’s test were performed to assess the publication bias. And the test showed our results were stable in the Asian subgroup.

Nevertheless, there are still some limitations that cannot be avoided in our meta-analysis. First, due to the lack of case-control studies related to nonsmokers, we failed to ascertain the association between the TNF-α-308 G/A variant and COPD risk in nonsmoking populations. Second, some unpublished studies might not be retrieved. Third, there were limited data to conduct subgroup analyses in Asians and Caucasians. The conclusions for TNF-α+489G/A and TNF-α -238G/A required more original studies to make them convincing.

Together, the present meta-analysis indicated that TNF-α-308G/A polymorphism was associated with an increased risk of COPD among Asians but not in Caucasians, and GA genotype carriers of TNF-α-308 had a higher risk of developing COPD compared to GG carriers when smoking status was not adjusted. However, in the meta-analysis with restrictions to smokers, no association was found between TNF-α-308 polymorphism and COPD susceptibility either in Asian smokers or Caucasian smokers. Moreover, the TNF-α+489G/A, -238G/A variants did not have an increased risk of COPD susceptibility.

## Data Availability

The original contributions presented in the study are included in the article/Supplementary Material, further inquiries can be directed to the corresponding author.
